# Improvement of the Activity of a Fungal Versatile-Lipase Toward Triglycerides: An *in silico* Mechanistic Description

**DOI:** 10.3389/fbioe.2019.00071

**Published:** 2019-03-29

**Authors:** Lucía Payá-Tormo, Javier Rodríguez-Salarichs, Alicia Prieto, María Jesús Martínez, Jorge Barriuso

**Affiliations:** Centro de Investigaciones Biológicas, Consejo Superior de Investigaciones Científicas, Madrid, Spain

**Keywords:** *Ophiostoma piceae*, lid hydrophobicity, molecular dynamics, *Candida rugosa*-like lipases, triglycerides

## Abstract

Some enzymes that belong to the *Candida rugosa*-like lipase family (abH03. 01) combine the activities of lipases and sterol esterases. Thus, they can act on water-insoluble carboxylic esters releasing long-chain fatty acids but also on sterol esters, although with different activity and affinity. The differences in the catalytic properties among the proteins of this family are explained by small changes in the hydrophobicity of some regions. One of such versatile enzymes is the sterol esterase/lipase from *Ophiostoma piceae* (OPE) that acts very efficiently on the two types of substrates. Structurally, OPE is characterized by the presence of a lid formed by a α-helix and two 3_10_-helices rich in hydrophobic amino acids. In this study, the *ope* gene was modified by directed mutagenesis in order to change specific amino acids in the lid region to modify its structure with the aim of increasing its hydrophobicity. Several recombinant forms of OPE were heterologously produced in *Pichia pastoris. In silico* molecular dynamics simulations have been used to decipher the mechanistic principles behind the improvements in substrate catalysis. The analyses suggested that the enhanced activity toward hydrophobic substrates such as triglycerides could be due to a better stabilization of the substrate in the lid region as a result of an increased hydrophobicity and an improved topology. These results indicate that *in silico* simulations can be useful for the optimization of the activity of lipases from the *C. rugose*-like family for different biotechnological applications.

## Introduction

Carboxylic ester hydrolases (EC 3.1.1) are a heterogeneous group of enzymes catalyzing the cleavage of ester bonds, including carboxylesterases (EC 3.1.1.1), triacylglycerol lipases (EC 3.1.1.3), and sterol esterases (EC 3.1.1.13). The natural role of lipases consists on catalyzing the hydrolysis of triglycerides to diglycerides, monoglycerides, free fatty acids and glycerol, while sterol esterases act on sterol esters releasing free sterols and fatty acids. Both types of enzymes have broad substrate specificity and can carry out hydrolysis reactions in aqueous media and synthesis reactions in the presence of organic solvents. Lipases are widely used in processes related to the food, detergents, cosmetics, pharmaceutical, textile, and paper industries among others (Morinaga et al., 2011; Singh and Mukhpadhyay, 2012) and have many biotechnological applications (Bornscheuer, 2002; Jaeger and Eggert, 2002). Although they are widespread in nature, being present from plants and animals to microorganisms, microbial lipases have gained special interest for industrial applications due to their stability, selectivity or wide substrate specificity (Loome and Senior, 1997; Ikeda et al., 2002; Brown et al., 2010). Some of the most commercially important lipases belong to yeasts, such as *Candida rugosa* and *Candida antarctica*, or filamentous fungi, such as *Aspergillus niger, Humicola lanuginosa, Mucor miehei*, and *Rhizopus* species (Jaeger and Reetz, 1998; Saxena et al., 1999).

In some occasions, the distinction between lipases and sterol esterases is not clear and enzymes initially described as sterol esterases have demonstrated to have activity also on triglycerides (Calero-Rueda et al., 2002; Kontkanen et al., 2006; Maeda et al., 2008). These enzymes, active toward acylglycerols and sterol esters (Vaquero et al., 2016), are classified under the *Candida rugosa*-like lipase family (abH03.01) and were recently denominated “versatile lipases” (Barriuso et al., 2016). Structurally, they display a α/β-hydrolase fold, with their catalytic machinery formed by a catalytic triad (serine, histidine and glutamic acid) and the oxyanion hole (Gutiérrez-Fernández et al., 2014) and a hydrophobic cavity covered by a mobile, amphipathic α-helix, named “lid” or “flap.” The difference in activity and substrate affinity toward triglycerides and sterol esters among the members of this family is explained by small changes in the hydrophobicity of these regions (Mancheño et al., 2003; Barriuso et al., 2013). Being considerably hydrophobic, these proteins tend to aggregate in dimers, tetramers, and even hexamers or more aggregated forms, displaying pseudoquaternary structures (Rúa et al., 1997; Pernas et al., 2001; Xiang et al., 2007).

Among others, the abH03.01 family includes the *O. piceae* sterol esterase (OPE) and the *C. rugosa* lipase isoenzymes that in spite of sharing more than 80% of sequence identity have different substrate affinity due to small divergences in the sequence of the lid region. The number of hydrophobic residues in the lid of the best characterized lipases produced by *C. rugosa* is higher in the isoforms that are more active on sterol esters than on triglycerides: Lip2 > Lip3 > Lip1 (12, 10, and 8 hydrophobic residues, respectively) (Lotti et al., 1994; Mancheño et al., 2003). Lip1 is the isoenzyme with the highest lipase activity. The substitution of the lid of Lip1 for that of Lip3 was sufficient to confer to Lip1 higher cholesterol esterase activity (Brocca et al., 2003). This suggests that the substrate specificity of these enzymes is related to the number of hydrophobic amino acid residues in the lid area. The *O. piceae* sterol esterase shows more than 40% sequence identity with *C. rugosa* lipases, but it contains more hydrophobic residues in the lid region, presenting high sterol esterase activity (Calero-Rueda et al., 2009).

In previous studies, a recombinant form of OPE was produced in *P. pastoris* at levels 7-fold higher than the native enzyme produced by *O. piceae* (Vaquero et al., 2015a). Furthermore, the characterization of the recombinant protein revealed its improved catalytic efficiency compared to the native one (Barba et al., 2012). The three-dimensional structures of OPE in its closed (OPE^c^) and its open conformations (OPE°) have also been reported by Gutiérrez-Fernández et al. (2014).

Considering the potential of these versatile enzymes in different biotechnological applications there is an increasing interest in the discovery and design of new catalysts from this family with modified substrate affinity (Barriuso et al., 2013; Barriuso and Martínez, 2015; Gupta et al., 2015). A strategy to achieve this objective is rational design, tailoring their catalytic properties to fulfill the industry needs by enzyme engineering based on structural-functional information, aided by computational simulations to reduce the experimental work. In this sense, *in silico* molecular simulations such as molecular docking and molecular dynamics have been used recently developed (van der Kamp and Mulholland, 2013). Here we have developed a computational protocol to determine the influence of amino acid substitutions in the specificity toward different substrates. We carried out a bioinformatics approach to improve the catalytic efficiency of OPE, expressed in *P. pastoris*, in hydrolysis reactions toward hydrophobic substrates for its potential biotechnological applications. We have focused in the lid region of the enzyme, trying to change its substrate affinity by replacing certain polar amino acids.

## Materials and Methods

### Reagents and Culture Media

All chemicals and reagents unless otherwise stated, were purchased from Sigma-Aldrich (Germany). Media were purchase from Fisher Scientific (Spain) unless otherwise stated.

### Construction of OPE Mutants

OPE mutants N81A, N94A and the double mutant, N81/94A, were constructed by site-directed mutagenesis. The mutation was introduced by PCR using the expression plasmid pPIC9OPE as template (Barba et al., 2012). The following primers, with the changed triplets underlined, were used: OPE N814 (Fw: 5′-CTGTCTACCGGCGCAGGCGA-3′ and Rv: 5′-TCGCCTGCGCCGGTAGACAG-3′); OPE N94A (Fw: 5′-AACCTGATCGCCATTCCCCT-3′ and Rv: 5′-AGGGGAATGGCGATCAGGTT-3′). In the case of the double mutant, OPE N81/94A, pPIC9OPE:N81A was used as template for mutagenic PCR using N94A primers. PCRs (50 μL final volume) were carried out in Mastercycler Pro S (Eppendorf) using 100 ng of template DNA, dNTP at 250 μM each, 0.25 μM of direct and reverse primers and 5 units of expand long template enzyme mix (Roche), in buffer 3. Reaction conditions were as follows: (I) 95°C for 1 min; (II) 18 cycles at 95°C for 50 s, 52°C for 50 s, and 68°C for 10 min; and (III) a final cycle at 68°C for 10 min. PCR products were treated with Dpn I restriction enzyme (Roche) to digest the parental strand. The result of the digestion was transformed in *E. coli* DH5α for propagation and plasmids extracted using High Pure Plasmid Isolation Kit (Roche).

*P. pastoris* KM71 strains were transformed with *Sal*I linearized plasmids following the manufacturer's protocol (Invitrogen, Carlsbad, CA, USA), and transformants were grown in YNB-His^−^ plates.

### Screening of OPE Mutants

*P. pastoris* transformed colonies were inoculated in 96-well plates with 50 μL YEPS medium (10 g/L yeast extract, 20 g/L peptone, 10 g/L sorbitol, 100 mM potassium phosphate buffer pH 6) and 0.5 % methanol (w/v) in each well. 75 μL of medium with methanol were added daily to maintain protein induction. The plate was incubated at 28°C and 250 rpm. After 3 days, the plate was centrifuged (5,000 g) and 10 μL of supernatant from each clone were dispensed in a new 96-well plate and assayed for esterase activity. 1.5 mM *p*-nitrophenyl-butyrate (pNPB) in 20 mM Tris-HCl buffer pH 7 was used as substrate, in a final volume of 200 μL (Calero-Rueda et al., 2009). The increase in absorbance was monitored at 410 nm for 5 min in a plate spectrophotometer (SpectraMaxPlus. BioNova). The two clones from each mutant with highest activity were inoculated in 250 mL flasks with 25 mL of expression medium to select the clone that secreted the maximal activity in flask.

### Protein Production and Purification

Protein was produced inoculating single colonies from the clone with the highest activity in 1 L flasks with 100 mL YEPS medium. Inoculation was done with 5 mL of fresh cultures grown overnight in YPD medium (10 g/L yeast extract. 20 g/L peptone. 20 g/L glucose). The flasks were incubated at 28°C and 250 rpm, where 0.5% methanol (w/v) was added daily for maintaining protein induction. Activity was checked in each flask until maximum activity was reached after 7–8 days.

The enzymes were purified as previously described (Vaquero et al., 2015b). Cells were harvested by centrifugation and supernatants concentrated to a volume of approximately 20–30 mL by ultrafiltration using a Millipore Pellicon TM−2 Miniholder (10 kDa cut-off membrane) and a micro-ultrafiltration system (Amicon-Ultra Centrifugal, Merck-Millipore, Darmstadt, Germany). Then, the enzyme was purified in a single hydrophobic chromatography step, using an AKTA FPLC system with a HiTrap Octyl Sepharose FF Cartridge (GE Healthcare). The sample and the column were equilibrated with 0.5 M (NH_4_)_2_SO_4_ and, after sample loading, a linear decreasing gradient from 0.5 to 0 M in buffer Tris-HCl 25 mM pH 7.0 was applied. OPE remained in the column at the end of the gradient and eluted by addition of 18 mM CHAPS in 25 mM Tris-HCl pH 7. The purified enzyme was dialyzed against 25 mM Tris-HCl, pH 7 to remove the detergent.

### Molecular Mass and Enzymatic N-Deglycosylation

The concentration of the pure protein was determined by the BCA bioassay (Thermo Scientific, Rockford, IL, USA) with bovine serum albumin as standard, and the apparent molecular mass estimated from 10% SDS-PAGE stained with Coomassie R-250. For N-deglycosylation with EndoH (Roche, Mannheim, Germany), the purified protein was dialyzed against sodium citrate buffer 50 mM pH 5.5, and then incubated with 250 mU of Endo H at 37°C for 24 h.

### Determination of the Kinetic Parameters

The apparent kinetic constants (*K*_m_ and *k*_cat_) of wild type (WT) OPE and the mutants were determined for the hydrolysis of the *p*-nitrophenol esters of different chain-length: pNPB, pNP-laurate (pNPL), and pNP-palmitate (pNPP). To calculate these parameters, the substrates' concentration was increased up to saturating values. A 20 mM stock solution of each substrate was prepared in acetone (HPLC grade). The assay mixtures (1 mL reaction volume) contained 2 mM substrate in 20 mM Tris-HCl pH 7 buffer and 1% (v/v) Genapol X-100, because the long-chain fatty acids substrates are not soluble in aqueous medium (Calero-Rueda et al., 2002). The increase of absorbance at 410 nm was monitored for 1 min at room temperature in a Shimadzu UV-1800 spectrophotometer with magnetic stirring. One unit of activity (1 U) is defined as the amount of enzyme releasing 1 μmol of *p*-nitrophenol (ε_410_ = 15.200 M^−1^·cm^−1^) per minute under the defined conditions (Gutiérrez-Fernández et al., 2014). Non-linear least-squares fitting of the experimental measurements were employed, adjusted to the Michaelis-Menten model, and calculated using the Sigma Plot 12.5 software (Systat Software Inc., San Jose, CA).

### Activity on Triglycerides

In order to check the activity on triglycerides of WT and OPE mutants, the hydrolysis of tripalmitin, as model compound, was assayed in aqueous medium. Stock solutions of the substrate and cholesten-3-one (internal standard for further chromatographic analysis) in acetone were first prepared. The necessary amounts to give final concentrations in the reaction mixture of 5 mM tripalmitin and 2.5 mM of the internal standard were dispensed in a clean vial. After evaporation in the vial they were resuspended in 20 mM Tris-HCl buffer pH 7 with 6 U/mL of enzyme, in a final volume of 100 μL. Reactions were performed at RT°C for 20 h under magnetic stirring, then dried by evaporation using a rotary evaporator at 60°C, and resuspended in chloroform. GC analyses were carried out in a 7890A gas chromatograph (Agilent, Palo Alto, CA) with the injector and FID set up at 350°C. Separation was done in a fused-silica capillary column SPB-1 (5 m x 250 μm × 0.25 μm, Supelco, Bellefonte. PA), using He (20 psi) as carrier gas. For quantification, the peak areas of palmitic acid and tripalmitin were normalized respect to the area of the cholesten-3-one internal standard.

### *In silico* Molecular Dynamics Simulations

Simulations were performed in the computer using the substrates mentioned above as ligands (pNPB, pNPL, pNPP, and tripalmitin). The enzyme structures utilized were that of the wild type OPE (pdb: 4BE9) and the same structure with the residues 81 and/or 94 substituted by alanine to simulate the OPE mutants N81A, N94A, and the double mutant N81/94A. The protein-ligand complexes were built using Maestro software (Schrödinger). The internal energies of the systems were minimized and guided to the nearest local minima. The energetic landscape of each protein-ligand complex was explored using Molecular Dynamics (MD) calculations. The MD trajectories were used to calculate the binding free energies (total and per residue). Our thermodynamics approximation was used to validate Molecular Dynamics results.

## Results and Discussion

To improve the catalytic properties of a given enzyme by rational design it is necessary to know its structure and catalytic properties. The versatile lipase OPE has been proposed for different biotechnological applications because of its high activity on triglycerides and esters of sterols (Barriuso et al., 2016). This protein belongs to the *C. rugosa*-like family and its crystal structure, which shows similarities with the lipases from *C. rugosa*, was recently elucidated (Gutiérrez-Fernández et al., 2014). *C. rugosa* lipase isoenzymes (CRL1-5) share over 80% sequence identity but diverge in the sequence of the lid, that modulates the access to the active site and affects its activity and substrate specificity (Brocca et al., 2003). With the aim of enhancing its affinity for triglycerides, the sequences of the versatile-lipases from *C. rugosa* CRL1-5 and OPE were aligned, and the arginine residues at positions 81 and 94 were selected for directed mutagenesis. These amino acids should not have structural function that would interfere in the catalytic activity of OPE, as they were situated in connecting loops that did not impede the formation of the α-helixes in the lid. Thus, two small, non-polar alanine residues replaced the two polar amino acids N81 and N94, since modification of the topology and hydrophobicity of the lid has been shown to be fundamental in the stabilization of highly hydrophobic and bulky substrates.

### OPE Mutants

The mutants were designed with the aim of increasing the efficiency of molecular recognition and the activity of OPE. The transformation efficiencies in *P. pastoris* KM71 were 72% for N81A, 67% for N94A and 94% for N81/94A. The clones with higher activity from each mutant were selected to produce the mutant proteins that were subsequently purified, measuring total activity against pNPB in each purification step (Table S1). The production levels obtained for the WT protein in this study are similar to those reported before (Barba et al., 2012). The molecular mass of the purified proteins, checked by SDS-PAGE, was around 75 kDa and the calculated N-glycosylation determined after treatment with EndoH was 28% (Figure S1), which is in agreement with Barba et al. (2012) and Calero-Rueda et al. (2009).

### Specific Activities

The activity of the purified proteins was assayed against *p*-nitrophenol esterified with fatty acids of different chain-length (pNPB, pNPL, and pNPP) as model substrates, and against tripalmitin as model triglyceride (Table 1). Compared with the WT protein, mutant N94A presented a significant increase in the activity against all the pNPs tested and tripalmitin, while N81A had an enhanced activity only against the long-chain substrates with an acyl chain of 16 carbon atoms (pNPP and tripalmitin). The double mutant N81/94A seems to be an intermediate between both single mutants, with higher activity against pNPL, pNPP and the triglyceride. These data showed how the specific activities of WT OPE and its mutants depend on the length of the hydrophobic chain of substrates; mutant N81A particularly increased its specific activities against longer substrates.

**Table 1:** Activity against different *p*-nitrophenol esters of WT and mutant variants of OPE.

		**Specific activity (U/mg)**			
**OPE**	**Protein concentration (mg/mL)**	**pNPB**	**pNPL**	**pNPP**	**Tripalmitin**
WT	1.83	116 ± 10	151 ± 22	102 ± 9	1.05 ± 0.12
N81A	2.27	121 ± 9	197 ± 43	171 ± 21	1.77 ± 0.10
N94A	2.78	181 ± 14	198 ± 14	129 ± 3	1.64 ± 0.05
N81/94A	3.34	132 ± 13	211 ± 22	158 ± 15	1.91 ± 0.08

*Activity against pNPs was calculated spectrophotometrically while activity against tripalmitin was calculated using gas-chromatography*.

### Apparent Kinetic Parameters

The kinetic values against pNP esters (Table 2) showed an increase in the overall catalytic efficiency (*k*_cat_/*K*_m_) of mutant N81A toward the long chain substrate (pNPP), while N94A and the double mutant are slightly more efficient against the short-chain substrate (pNPB). Concerning the middle-length chain substrate, pNPL, the WT protein maintained a catalytic efficiency close to N81A or superior to the two other mutants. In this case, N81A displayed a significantly increased affinity toward pNPL than the WT OPE, but the turnover against this substrate was lower. In the case of N94A, there is an improvement in the *k*_*cat*_ against pNPP but its *K*_m_ value is significantly higher. Concerning pNPL, the *k*_*cat*_ value calculated for WT was higher than those from the other lid variants. When comparing the kinetic values of OPE mutants with those from other lipases from the *C. rugose*-like family such as Aspni5, Necha2 and Trire2, it can be observed that the enzymes with higher number of hydrophobic residues in the lid (Necha2 and Trire2) showed higher catalytic efficiency toward pNPL and pNPP, respectively (Vaquero et al., 2015b). These results together indicate that the hydrophobic amino acids substitutions in the lid may have effect in the activity of the enzyme depending on their position.

**Table 2:** Kinetic parameters of the different OPE variants obtained in this work, calculated for pNPB, pNPL and pNPP.

		**Substrates**		
**OPE**	**Parameter**	**pNPB**	**pNPL**	**pNPP**
WT	*K*_m_	0.26 ± 0.08	1.1 ± 0.2	0.24 ± 0.1
	*k_*cat*_*	146.9 ± 12.1	600.1 ± 48.7	103.3 ± 10.9
	*k_*cat*_*/*K*_m_	567.5 ± 142.6	552.6 ± 73.2	434.4 ± 125.5
N81A	*K*_m_	0.22 ± 0.1	0.58 ± 0.3	0.2 ± 0.2
	*k_*cat*_*	142.5 ± 16.6	253.7 ± 36.5	162.0 ± 48.9
	*k_*cat*_*/*K*_m_	647.5 ± 158.3	435.1 ± 142.5	806.1 ± 177.4
N94A	*K*_m_	0.19 ± 0.08	1.1 ± 0.2	1.1 ± 0.7
	*k_*cat*_*	158.1 ± 24.0	315.1 ± 20.9	216.6 ± 60.8
	*k_*cat*_*/*K*_m_	814.8 ± 184.8	286.9 ± 118.4	204.3 ± 83.3
N81/94A	*K*_m_	0.17 ± 0.08	0.85 ± 0.4	0.35 ± 0.2
	*k_*cat*_*	160.6 ± 15.9	252.67 ± 41.6	124.1 ± 20.9
	*k_*cat*_*/*K*_m_	944.7 ± 195.7	297.26 ± 112.3	350.5 ± 111.8

*The data reported for the wild type OPE by Vaquero et al. (2015b) have been included*.

### Molecular Dynamics Simulations

**Figure 1:**
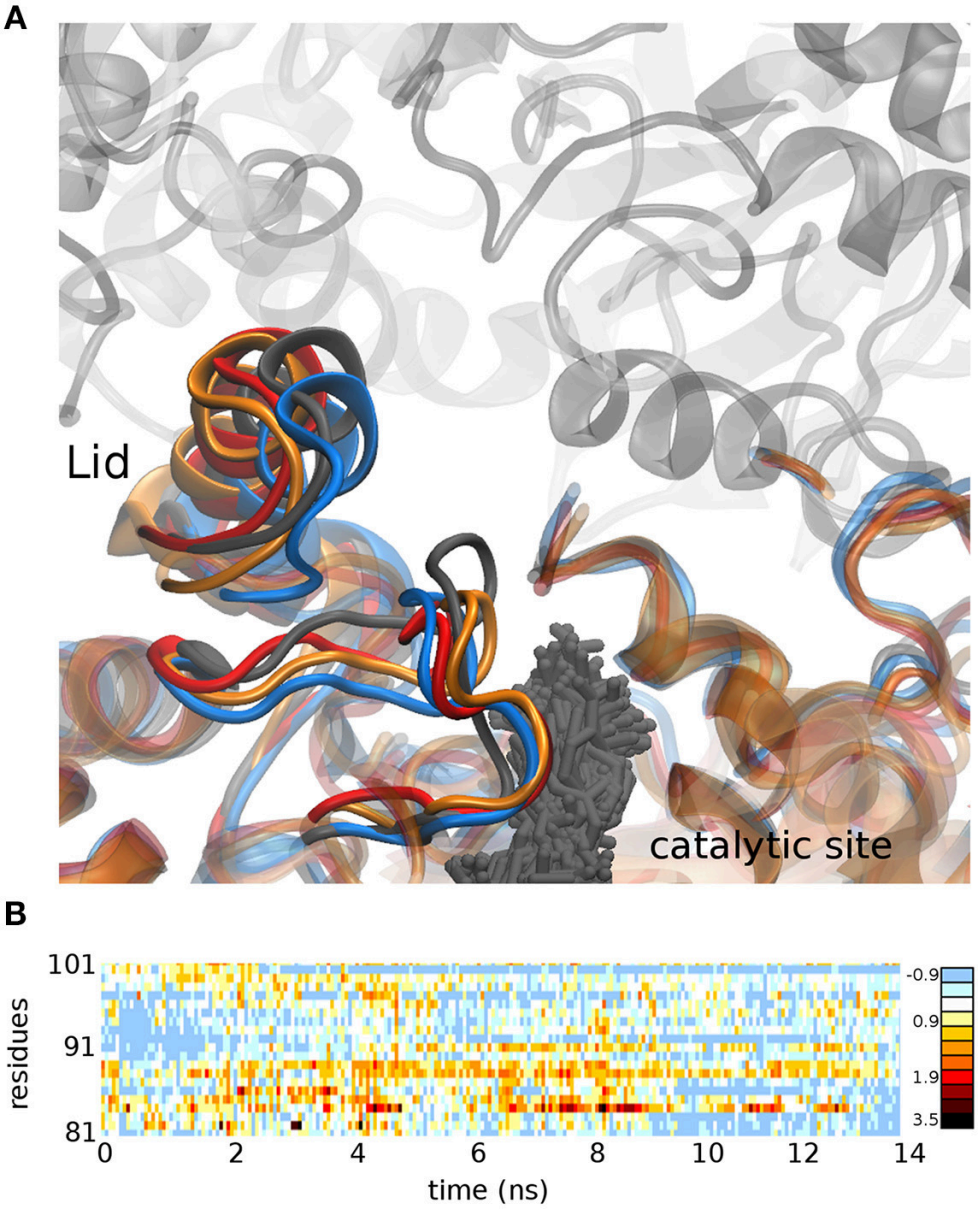
**(A)** Conformation adopted after 15 ns molecular dynamics simulation in the WT OPE (gray), mutant N81A (blue), mutant N94A (red), and the double mutant N81/94A (orange). The positions adopted by the substrate pNPB in all the dynamics of the WT protein are shown (gray sticks). **(B)** Difference between the RMSD of mutant N81A and N94A in the lid region (residues 81-101) in a 15 ns molecular dynamics simulation. Positive values represent greater movement in the mutant N81A, while negative values represent greater movements in the mutant N94A.

To get insight into the mechanistic basis of the improved catalytic activities, docking and molecular dynamics experiments were performed. We proceeded to analyze the geometrical and physical-chemical characteristics of the new lid area based on CASTp methodology (Tian et al., 2018) and in the Chimera package (Pettersen et al., 2004). The results in Figure 1 show how N81A mutant and the double mutant N81/94A presented a modified flexibility in the lid region compared to N94A mutant and WT OPE. The modification of residue 81 in OPE avoided the establishment of an anchor H-bond between N81 and H272 (distance in PDB 4BE9 of 3.3 Å) that probably participated in the stabilization of the open form of OPE. This conformational modification is transferred to the loop 306-316 that is forming part of the active site, thus modifying the binding of the substrate. Moreover, the flexibility in the lid region of the double mutant seems to be intermediate between the N81A and N94A mutants. However, the increase in the lid flexibility in N81A did not affect much the affinity or catalysis of OPE against the short-chain substrate.

**Figure 2:**
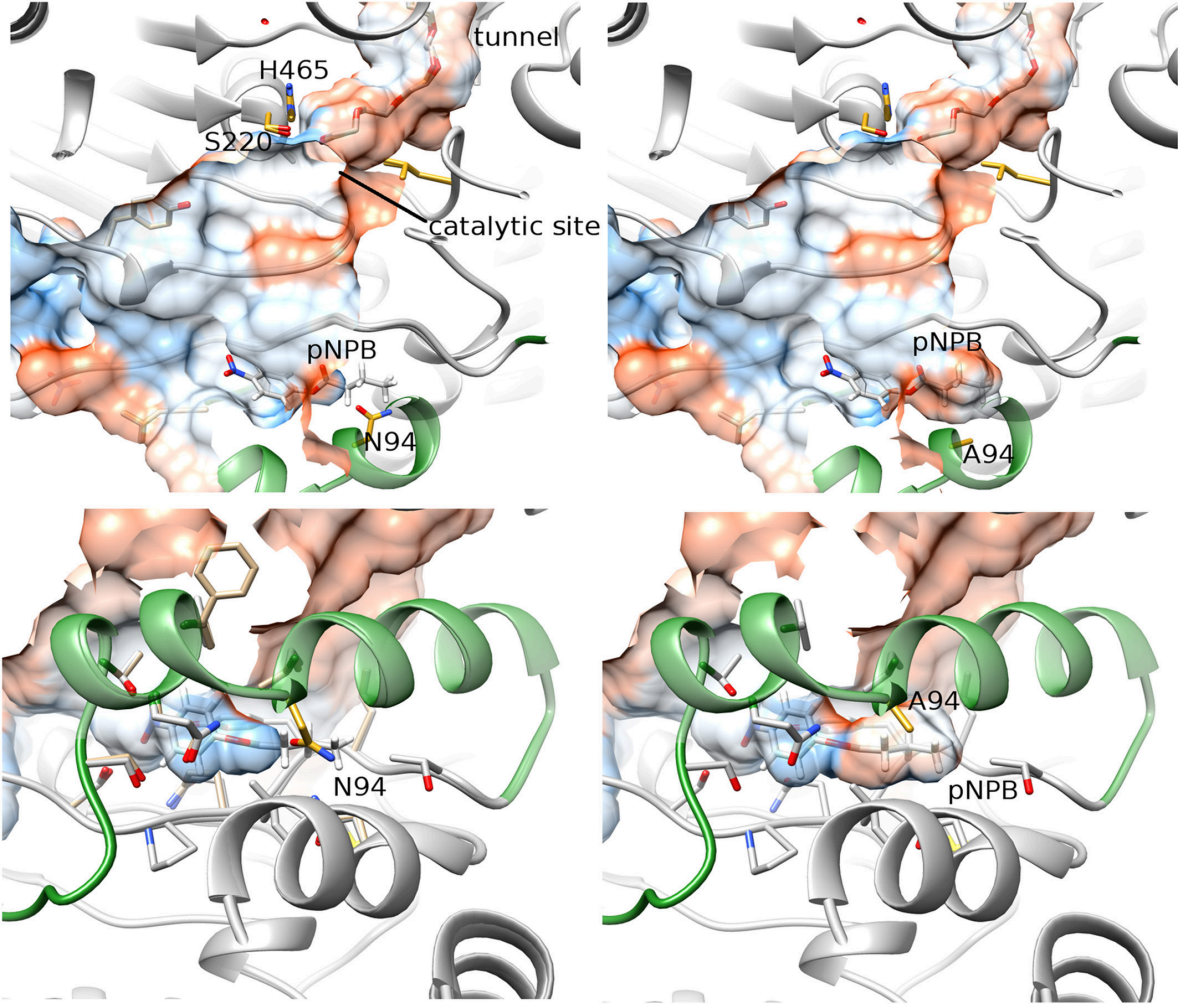
Representation of the docking between pNPB and OPE WT (left) and N94A (right). The lower panels represent a rotated view of the lid area. The hydrophobicity of surfaces is represented from blue for the most hydrophilic, to red for the most hydrophobic regions. The lid is represented in forest green.

On the other hand, mutant N94A presented a larger cavity in the lid area (Figure 2, right) than that in the WT protein (Figure 2, left). The substitution of the arginine 94 for alanine led to the formation of a pocket with a volume of 99 Å^3^, able to accommodate a pNPB molecule. Furthermore, the hydrophobicity calculated in this region showed an increase in surface of 115 Å^2^ (Figure 2). These changes in the topology and hydrophobicity of the lid region would allow a better stabilization of the hydrophobic part of the ligand that remains outside the enzyme during catalysis, and a better accommodation of the substrates oriented toward the catalytic active site (Figures 1A,2)

**Figure 3:**
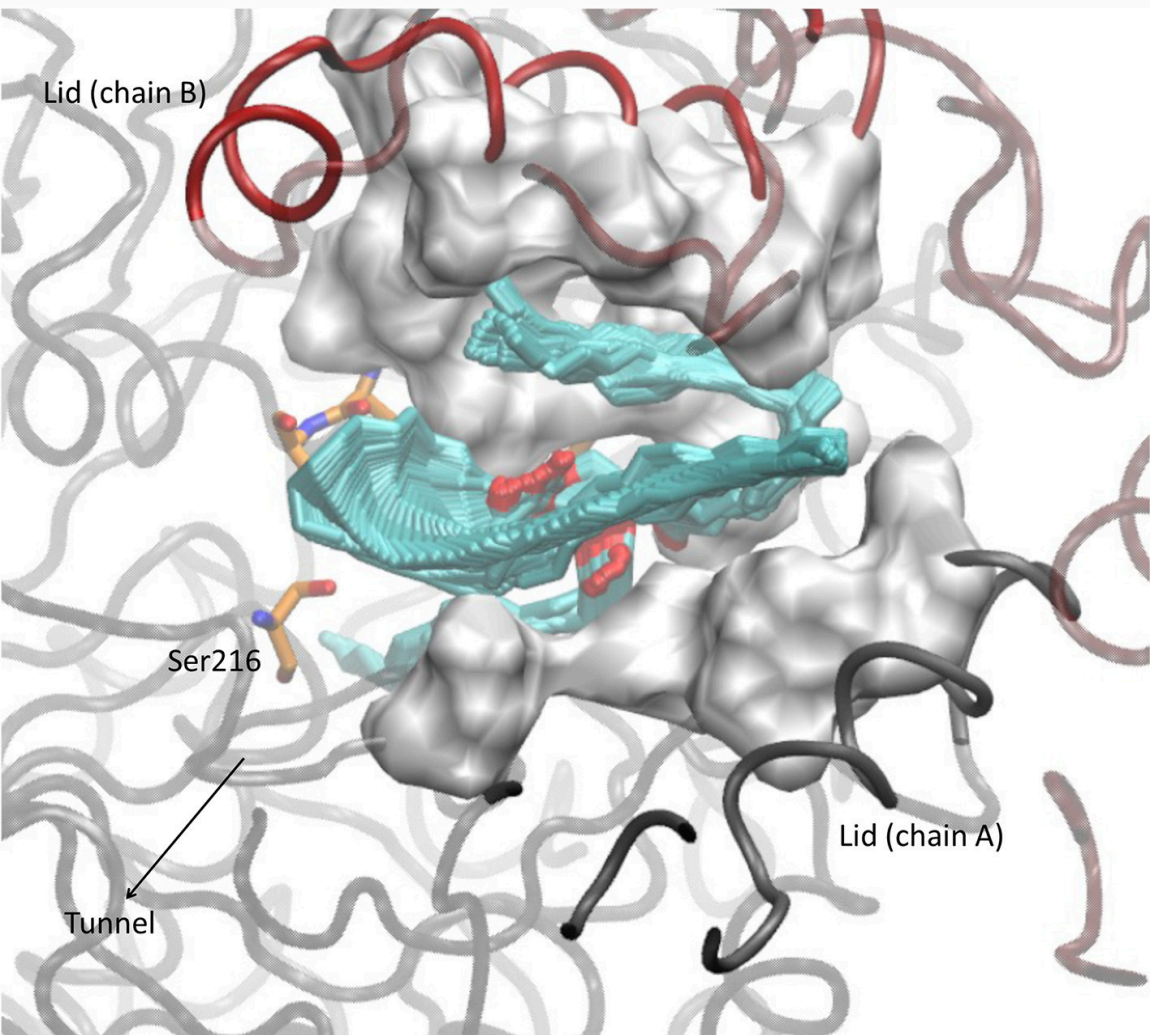
Conformation adopted after 15 ns molecular dynamic simulation in the WT OPE docked with a molecule of tripalmitin.

Tripalmitin hydrolysis was studied to evaluate the activity of the mutants toward a more hydrophobic substrate (Table 1). The correlation between the number of hydrophobic amino acids in the lid region with the affinity toward triglycerides has been previously reported (Mancheño et al., 2003; Dominguez et al., 2006; Barriuso et al., 2013). The drawing in Figure 3 shows how the hydrophobic diglyceride that remains outside the enzyme interacts with the lid region, and thus how the increase of hydrophobicity and flexibility in the lid could provide a better interaction and stabilization of the substrate.

In conclusion, we have constructed three mutants of the versatile-lipase OPE. These mutants aimed to improve the catalysis of hydrophobic substrates by increasing the hydrophobicity of the lid region. The mutations performed generally had a positive impact on the catalysis of pNP substrates and tripalmitin, in agreement with the data from other authors that have shown that the lid region is fundamental for the substrate recognition.

The double mutant did not show a synergist effect in its activity compared to the single mutants. However, it combined the catalytic properties of the single mutants, which displayed structural changes in the lid region and the catalytic site.

Molecular dynamics simulations have shown that besides a more hydrophobic lid, the N94A mutant had a cavity in this area that could stabilize small substrates such as pNPB acting as a molecular trap. Further studies using sterol esters and other triglycerides will be performed to gain knowledge about the influence of these mutations on substrates' affinities. Current *in silico* protein modeling technologies can help to design efficient biocatalysts with industrial applications; however, experimental studies are necessary in order to verify these changes in substrate catalysis, as well as the structural integrity of the enzyme.

## Data Availability

All datasets generated for this study are included in the manuscript and/or the Supplementary Files.

## Author Contributions

LP-T performed the experimental work. JR-S performed the computational calculations. AP participated in the interpretation of the results and preparation of the manuscript. JB and MM designed the work and participated in the interpretation of the results and preparation of the manuscript.

### Conflict of Interest Statement

The authors declare that the research was conducted in the absence of any commercial or financial relationships that could be construed as a potential conflict of interest.

## Supplementary Material

Figure S1 SDS-PAGE of purified OPE incubated with Endo H. Lane 1: molecular mass marker. Lanes 2, 4 and 6: OPE:N81A, N94A and N81/94A, respectively, incubated with EndoH. Lanes 3, 5 and 7: OPE:N81A, N94A and N81/94A, respectively, without EndoH treatment. The band around 25 kDa corresponds to EndoH.

Table S1 Activity and total volume along purification of native and OPE variants.
