# Supplementary figures and images for: Improvement of the Activity of a Fungal Versatile-Lipase Toward Triglycerides: An in silico Mechanistic Description
Source: Front Bioeng Biotechnol. 2019 Mar 29;7:71. doi: 10.3389/fbioe.2019.00071 (PMC6449727; doi:10.3389/fbioe.2019.00071)

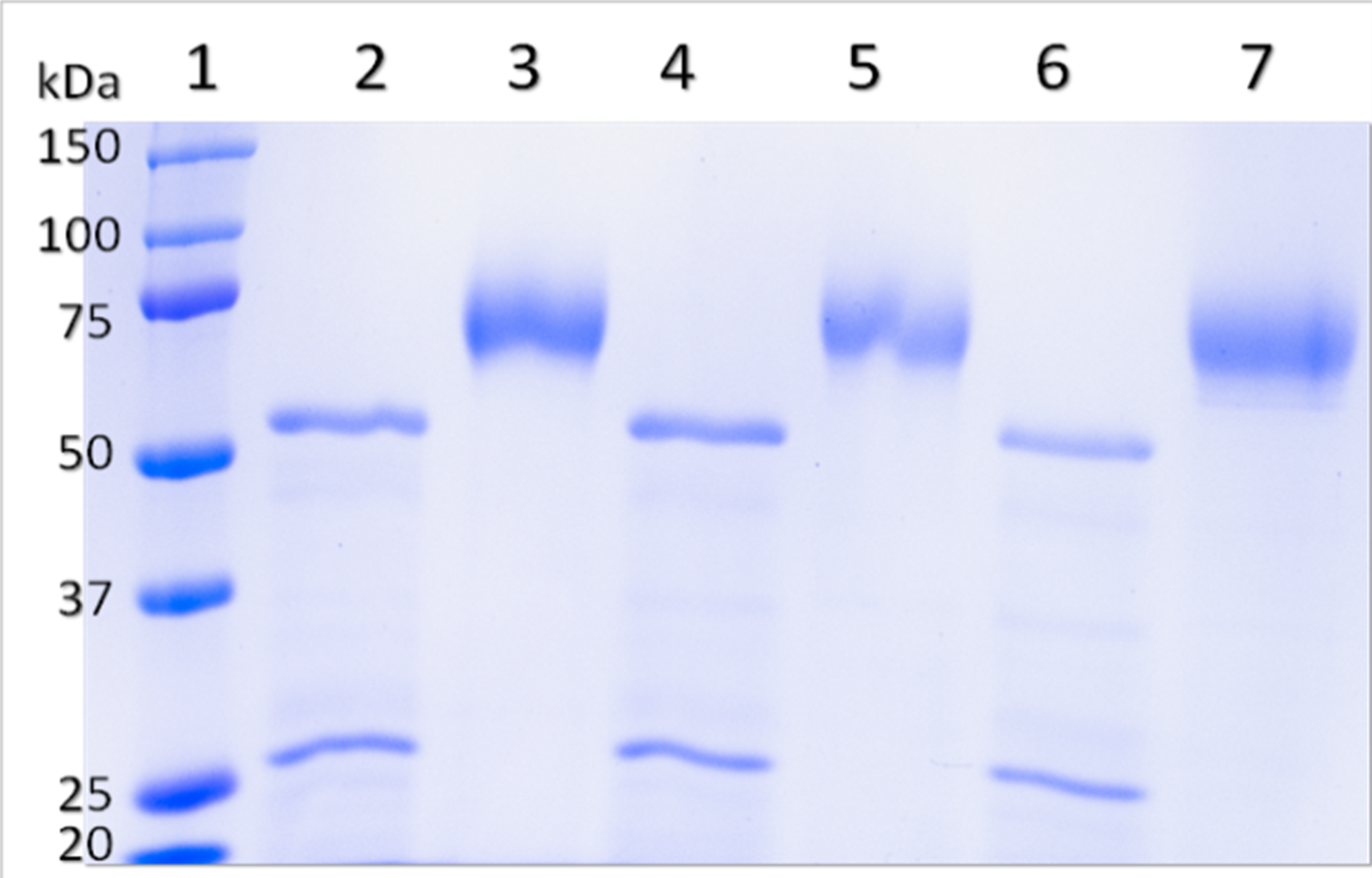

Supplement: Figure S1 — SDS-PAGE of purified OPE incubated with Endo H. Lane 1: molecular mass marker. Lanes 2, 4 and 6: OPE:N81A, N94A and N81/94A, respectively, incubated with EndoH. Lanes 3, 5 and 7: OPE:N81A, N94A and N81/94A, respectively, without EndoH treatment. The band around 25 kDa corresponds to EndoH. [file Image_1.TIF]
