# Supplementary material for: Improvement of the Activity of a Fungal Versatile-Lipase Toward Triglycerides: An in silico Mechanistic Description
Source: Front Bioeng Biotechnol. 2019 Mar 29;7:71. doi: 10.3389/fbioe.2019.00071 (PMC6449727; doi:10.3389/fbioe.2019.00071)
Supplement: Table S1 — Activity and total volume along purification of native and OPE variants. [file Table_1.docx]

**Supplementary material**

Table SI. Activity and total volume along purification of native and OPE variants.

| **Step** | **Total activity against pNPB (U)** | | | | | **Total volume (mL)** | | | | |
| --- | --- | --- | --- | --- | --- | --- | --- | --- | --- | --- |
|  | WT | N81A | N94A | N81/94A | WT | | N81A | N94A | N81/94A |  |
| **Supernatant culture** | 1450 | 1728 | 2774 | 2942 | 100 | | 380 | 780 | 880 |  |
| **Filtron concentrate** | N/A* | N/A | 2960 | 3355 | N/A | | N/A | 250 | 200 |  |
| **Amicon concentrate** | 1590 | 1369 | 2460 | 2648 | 40 | | 30 | 25 | 25 |  |
| **Octyl-Sepharose** | 404 | 101 | 952 | 2013 | 10 | | 10 | 10 | 10 |  |

*Not applicable. This step was not necessary.
